# Medical History from the Earliest Times

**Published:** 1892-06-18

**Authors:** E. T. Withington


### Medical History from the Earliest Times.

By E. T. Withington, M.A., M.B. Oxon.

### XIII.—THE ALEXANDRINE ANATOMISTS.

When Ptolemy I. established the "Museum" of Alexandria, about B.C. 300, he made that city the centre of Greek science, including medicine, a position which it maintained for nearly a thousand years, so that, even in the fourth century A.D., to have studied at Alexandria was a sufficient recommendation for a young physician in any part of the Roman Empire.

The earliest members of the school, Herophilus and Erasistratus, were among the greatest it produced, and they carried the science of human anatomy to the highest point then attainable. There are, indeed, traces of this study in older times, but these may safely be dismissed in the language which Thucydides applies to the deeds of the earlier Greeks, "Little is known of them, but they were probably no great things." Nor is it necessary to revive the discussions, dating, perhaps, from classical times, as to which particular discoveries are to be attributed to each of the great anatomists. Both investigated the nervous system, traced the origin of the nerve trunks to the brain and spinal cord, and distinguished sensory and motor branches, though they perhaps sometimes mistook tendons for the latter. Both described the coverings of the brain, and Herophilus traced the sinuses of the "dura mater" to their meeting point, which is still known by the name he gave it, the "wine press," or "torcular" Herophili. He also gave an account of the ventricles of the brain, especially the fourth, with its "calamus scriptorius," and believed, like some modern physiologists, that it was the special seat of the soul. Both seem to have noticed the lacteals, for Erasistratus says that the mesenteric arteries sometimes contain milk instead of "vital spirits," and Herophilus asserts that some veins of the mesentery end, not in the portal vein, but in glands. The latter also described and named the hyoid bone, the duodenum, and the prostate gland, and made a very careful study of the eye, thereby greatly improving the old operation for cataract, though the assertion that he first extracted the lens instead of merely depressing it seems unwarranted. Erasistratus investigated the anatomy of the heart, with its valves and chordæ tendineæ, while Herophilus is said to have made the first *post-mortem* examinations, at some of which King Ptolemy himself was present.

Both were physicians as well as anatomists, and here they differed widely. Herophilus maintained the humoral pathology, and revered Hippocrates, in so much that when obliged to contradict him he always avoided mentioning his name. His tutor, Praxagoras, had first shown the importance of the pulse, to which Herophilus devoted great attention, comparing the different varieties to the rhythms of music, and giving them special names, one of which, the leaping or goat-like pulse (*pulsus caprizans s. dicrotus*) still survives. He also wrote on the causes of sudden death, attributing it to paralysis of the heart, and noticed a case of its occurrence during the extraction of a tooth. He put a high value on drugs, which he called "the hands of the gods," and used them in great variety. When asked what was the best quality of a physician, Herophilus is said to have replied, "The power to distinguish the possible from the impossible."

Erasistratus, on the contrary, rejected the humoral doctrines, and founded a system of his own, based on the theory that the arteries contain air or "vital spirits." To meet the obvious objection that these vessels bleed when injured, he assumed the existence of communications between them and the veins, closed normally, but allowing blood to enter the arteries as soon as the air escapes. Noticing that wounds are often followed by inflammation, he assumed that this also was due to a passage of blood into the arteries, and that when it involved the larger vessels it produced fever. The great cause of inflammation, fever, and disease generally was, he considered, an overfulness of the veins, or "plethora," which compelled some of the blood to pass through into the arteries. Yet, strangely enough, he entirely rejected bleeding, treating his patients by low diet, and by bandaging their limbs with the view of closing the communications by pressure. This horror of venæsection he probably acquired from his teacher, Chrysippus, of Cnidus, who had spent sixteen months with the Egyptian priests, and had learnt from them the doctrine that "the blood is the life," or, as Chrysippus called it, "the food of the soul." His medicines were of the mildest character, consisting of laxatives, barley water, and wine, which last he gave in homœopathic doses, beginning with three drops and gradually increasing. This, however, was not due to any timidity, if we may believe the story that Erasistratus was in the habit of cutting down upon the liver and spleen, and applying his drugs directly to the surface of those organs. He rejected the operation of tapping the abdomen in dropsy, for he declared it did not affect the origin of the disease, which was usually an affection of the liver, a fact which he may have discovered by means of *post mortem* examinations. Finally, Erasistratus invented a catheter, though probably not the first instrument of that nature. The followers of both the great anatomists formed "schools," which lasted more than three centuries, and Galen directs two of his treatises against the Erasistrateans and their anti-venæsection principles.

The development of anatomy would naturally be followed by improvements in surgery, and that this was the case may be seen by comparing the condition of the art described by Celsus with that found in the Hippocratic writings. The advance of mechanical knowledge tended in the same direction, and the wonderful inventions by which Archimedes defended Syracuse against the Romans were more successfully employed by the surgeons of Alexandria. The endless screw proved especially useful, while the "trispaston," a contrivance for dragging ships on shore, was adapted by Pasicrates to the reduction of dislocations, and was soon to be found in every respectable gymnasium. The learned leisure of the medical occupants of the Museum was further employed in devising new and complicated modes of bandaging, and the majority of our existing methods are probably only survivals of the fittest of those then invented.

The various operations introduced at this time will be more conveniently discussed when we come to the golden age of Greek surgery, the days of Antyllus, Heliodorus, and Archigenes, but, in conclusion, we must not shrink from examining a painful and discreditable aspect of the Alexandrine medicine. It is said that the Ptolemies, in their zeal for science, handed over condemned criminals to Herophilus and Erasistratus, and that the latter opened the various cavities of their yet living bodies, in the hope of making important physiological discoveries. Modern writers have attempted, though with scanty success, to discredit this story; antiquity never doubted it. Celsus, while condemning the practice, adduces arguments to defend it, but we may well hope that Tertullian exaggerates when he puts the number of the victims at six hundred. Nor are we without somewhat similar instances in more modern times. About the year 1550 the Duke of Tuscany handed over a condemned criminal to the medical faculty of Pisa, "to kill after their own fashion, and anatomise." Fallopius gave the unfortunate man two large doses of opium, but it may be questioned whether anyone would have interfered, even had he followed the supposed example of Herophilus. Were not tortures equally great daily inflicted in the names of Law and Religion? The same stories have been told of great artists, Parrhasius and Michael Angelo, who are said to have tried in this way to obtain models of the suffering Prometheus, and—*horribile dictu!*—the crucified Saviour. Such tales, however, might readily be manufactured, and we may perhaps still venture to hope that they are all equally false; but there can be no doubt that experiments of the effects of poisons and antidotes were frequently made upon condemned criminals by Greeks, Arabs, and even Christians.

